# Polar electrostatic forces drive poleward chromosome motions

**DOI:** 10.1186/s13008-014-0005-3

**Published:** 2014-12-30

**Authors:** Lucian John Gagliardi, Daniel H Shain

**Affiliations:** Department of Physics, Rutgers The State University of New Jersey, Camden, NJ 08102 USA; Department of Biology, Rutgers The State University of New Jersey, Camden, NJ 08102 USA

## Abstract

Recent experiments revealing nanoscale electrostatic force generation at kinetochores for chromosome motions have prompted models for interactions between positively charged molecules in kinetochores and negative charge at and near the plus ends of microtubules. A clear picture of how kinetochores and centrosomes establish and maintain a dynamic coupling to microtubules for force generation during the complex motions of mitosis remains elusive. The molecular cell biology paradigm requires that specific molecules, or molecular geometries, for polar force generation be identified. While progress has been made regarding explanations of kinetochore-based chromosome motility, molecular machinery for chromosome poleward movements at centrosomes has yet to be identified. The present work concerns polar generation of poleward force in terms of experimentally known electric charge distributions at microtubule minus ends and centrosomes interacting over nanometer distances.

## Introduction

Current thought on mitotic motions is being considered in a more electrostatics-based framework [1], corroborating theoretical predictions made a decade ago [2,3]. Chromosome movement depends on kinetochore-microtubule dynamics: a chromosome can move toward a pole only when its kinetochore is connected to microtubules emanating from that pole [4]. Microtubules continually assemble and disassemble, so the turnover of tubulin is ongoing. The characteristics of microtubule lengthening (polymerization) and shortening (depolymerization) follow a pattern known as “dynamic instability”: *i.e.*, at any given instant some of the microtubules are growing, while others are undergoing rapid breakdown. In general, the rate at which microtubules undergo net assembly – or disassembly – varies with mitotic stage [5]. Here we propose that nanoscale electrostatic interactions between microtubule minus ends and charge distributions at centrosomes are responsible for polar generation of force for poleward chromosome motility during mitosis.

**Figure 1:**
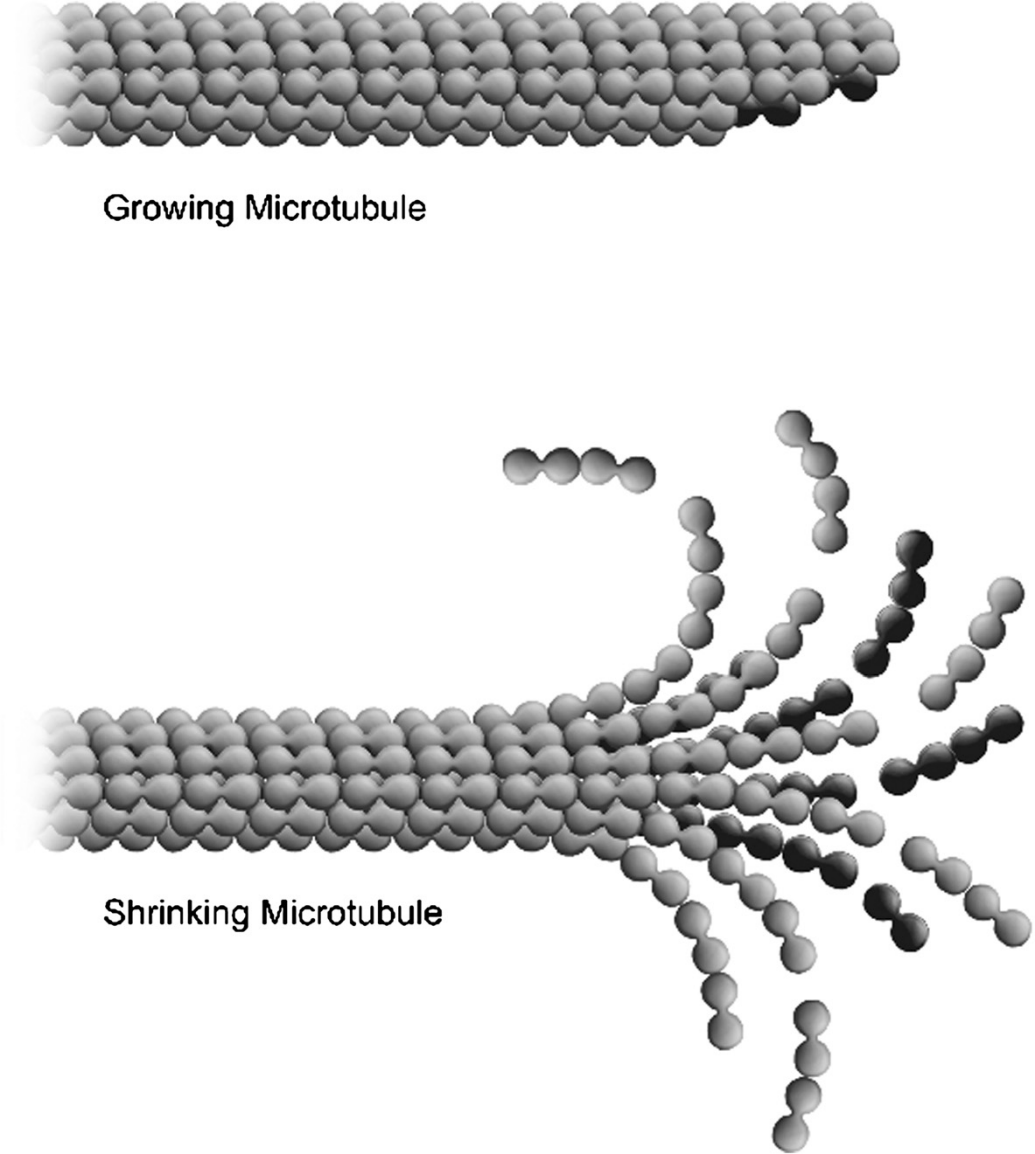
**Shrinking (showing protofilament curling) and growing microtubules.**

Electrostatic force generation at kinetochores by a similar mechanism is described elsewhere [3,6]. Kinetochore minus-end disassembly at poles associated with poleward microtubule flux is known to produce a force that can do work [7]. Specifically, taxol-induced mitotic spindle shortening (i.e., inhibition of microtubule assembly/disassembly at kinetochores) occurs by minus-end disassembly simultaneously generating a poleward force that is sufficient to stretch centromeric chromatin between sister kinetochores as much as it is stretched in control metaphase cells [7]. These experiments demonstrate a fundamentally different mechanism for polar force generation in the context of poleward chromosome motility. Some have proposed that minus-end microtubule disassembly is mediated by microtubule motors like Kar3 [8] or cytoplasmic dynein [9], while other older models postulate a mechanism for increasing the lability of microtubule minus ends [10]. More recently, coupling molecules and molecular structures have been suggested to convert the progressive splaying (arching out into a “ram’s horn” configuration) of disassembling microtubule protofilaments (see Figure 1) into poleward force generation for chromosome movements. In later versions of these models, the splaying tendency of GDP-tubulin protofilaments to curve in this manner provides a “power stroke” that pulls on centromeric chromatin through kinetochore fibrils [11]. Other models utilize ring-like coupling kinetochore Dam1/DASH complexes for this coupling [12]. Ndc80 is another kinetochore-based molecule that has been implicated in poleward force generation [1]. Importantly, none of these molecules or proposed mechanisms occur at poles for coupling to splaying protofilaments – or to minus-ends of microtubules in any way – for poleward force production at centrosomes (poles).The model proposed here is restricted to polar generation of poleward force for chromosome motility, an aspect of mitosis for which there is no established molecular biology paradigm.

## Discussion

### Cellular electrostatics

In the cytoplasmic medium (cytosol) within biological cells, electrostatic fields are subject to strong attenuation by screening with oppositely charged ions (counterion screening), decreasing exponentially to much smaller values over a distance of several *Debye lengths*. The Debye length within cells is typically of order 1 nm [13], and since eukaryotic cells have much larger dimensions, one would be tempted to conclude that electrostatic force is not a major factor for mitotic chromosome movements in biological cells. However, the presence of microtubules, as well as other factors discussed below, change this concept completely.

Microtubules can be considered as intermediaries that extend the reach of the electrostatic interaction over cellular distances, making the second most potent force in the universe available to cells in spite of their ionic nature. Microtubules are 25 nm diameter cylindrical structures comprising *protofilaments*, each consisting of tubulin dimer subunits, 8 nm in length, aligned end-to-end, parallel to the microtubule axis. The protofilaments are bound laterally to form a cylindrical microtubule, which has a similar structure in all eukaryotic cells. Cross sections reveal that a microtubule wall comprises a circle of 4 to 5 nm diameter subunits and typically contains 13 subunits, as observed *in vivo*. Neighboring dimers along protofilaments exhibit a small (B-lattice) offset of 0.92 nm from protofilament-to-protofilament.

Experimental differences have been observed in profiles of growing and shrinking microtubules, as depicted in Figure 1. A number of investigations have focused on the electrostatic properties of microtubule tubulin subunits [14-17]. Large scale calculations of the tubulin molecule have been carried out using molecular dynamics programs along with protein parameter sets. The dipole moment of tubulin has been calculated to be as large as 1800 Debye (D) [15,18]. Experiments [19] have shown that tubulin net charge depends strongly on pH, varying quite linearly from −12 to −28 (electron charges) between pH 5.5 and 8.0. This may be important for tubulin electrostatics during mitosis because a number of cell types exhibit a decrease of 0.3 to 0.5 pH units from a peak at prophase [20].

Tubulin has a large overall negative charge of 20 at pH 7, and as much as 40% of this charge resides on the C-termini [21]. C-termini can point nearly perpendicularly outward from the microtubule axis as a strong function of pH_i_, extending 4–5 nm at pH_i_ 7 [21], and can exist in at least 2 other conformational states where they bind to the microtubule surface at lower pH_i_ [22].

The pH_i_ in the vicinity of the negatively-charged centrosome will be lower than the overall pH_i_, due to the negative charge. This pH lowering in the vicinity of negative charge distributions is a fundamental principle; intracellular pH in such limited volumes is often referred to as *local* pH. As one might expect from classical Boltzmann statistical mechanics, the hydrogen ion concentration at a negatively-charged surface is the product of the bulk phase concentration and the factor e^−*eζ/kT*^ , where *e* is the electronic charge, *ζ* is the (negative) potential at the surface, and *k* is Boltzmann’s constant [23]. For example, for typical mammalian cell membrane negative charge densities, and therefore typical negative cell membrane potentials, the local pH can be reduced 0.5 to 1.0 pH unit.

Experiments have revealed that mitotic spindles can assemble around DNA-coated beads incubated in *Xenopus* egg extracts [24]. Since the phosphate groups of the DNA manifest a net negative charge at the pH of this experimental system, the pericentriolar material (*i.e*., the *centrosome matrix* within which the microtubule dimer dipolar subunits assemble in many cell types to form asters [25]) was proposed to carry a net negative charge [2,26]. Centrosomes have subsequently been shown to carry a net negative charge by direct measurement [27].

Thus given the electric dipole nature of microtubule subunits and the efficiency of aster self-assembly, it is likely that microtubule *minus* ends proximal to centrosomes are positively-charged, with *plus* free ends negatively-charged. These assignments of net charge at microtubule free ends are consistent with (1) large scale calculations of tubulin dimer subunits showing that 18 positively-charged calcium ions are bound within *β* monomers with an equal number of negative charges localized at adjacent *α* monomers [14,15], and experiments revealing that microtubule plus ends terminate with a crown of *α* subunits and minus ends terminate with *β* subunits [28]; (2) the lower local pH vicinal to a negatively-charged centrosome matrix will cause a greater expression of positive charge on free microtubule minus ends; and (3) negative charges on centrosome matrices will induce positive charges on microtubule minus ends.

Apart from the ability of microtubules to extend electrostatic interactions over cellular distances, the range of electrostatic fields within the cytosol itself is longer than ordinary counterion screening considerations would dictate. One can reasonably expect that the electric dipole nature of tubulin subunits greatly assists their self-assembly into the microtubules of the asters and spindle. Thus we may envision that electrostatic fields organize and align the electric dipole dimer subunits, thereby facilitating their assembly into microtubules that form the asters and mitotic spindle [26]. This self-assembly would be aided by reduced counterion screening due to layered water adhering to the net charge of the dipolar subunits. Such water layering to charged proteins has long been theorized [29,30], and has been confirmed experimentally [31]. Additionally, layered water between sufficiently close charged proteins has a dielectric constant that is considerably reduced from the *bulk* value distant from charged surfaces, further increasing the tendency for an electrostatic enhancement of aster and spindle self-assembly. The parameters defining “sufficiently close” charged molecular surfaces are addressed below.

The combination of these two effects (or conditions)--water layering and reduced dielectric constant--can significantly influence cellular electrostatics in a number of important ways related to cell division. It is convenient in the present work to characterize gaps between charged surfaces within cells that allow these two effects to significantly enhance electrostatic interactions, as *critical gaps* or *critical distances*. These two conditions for charged molecular surfaces at close range have important consequences regarding electrostatic polar force generation for poleward chromosome motion during mitosis. Electrostatic poleward force generation at kinetochores by a similar mechanism is described elsewhere [3,6].

### Electrostatic microtubule poleward disassembly force at cell poles

**Figure 2:**
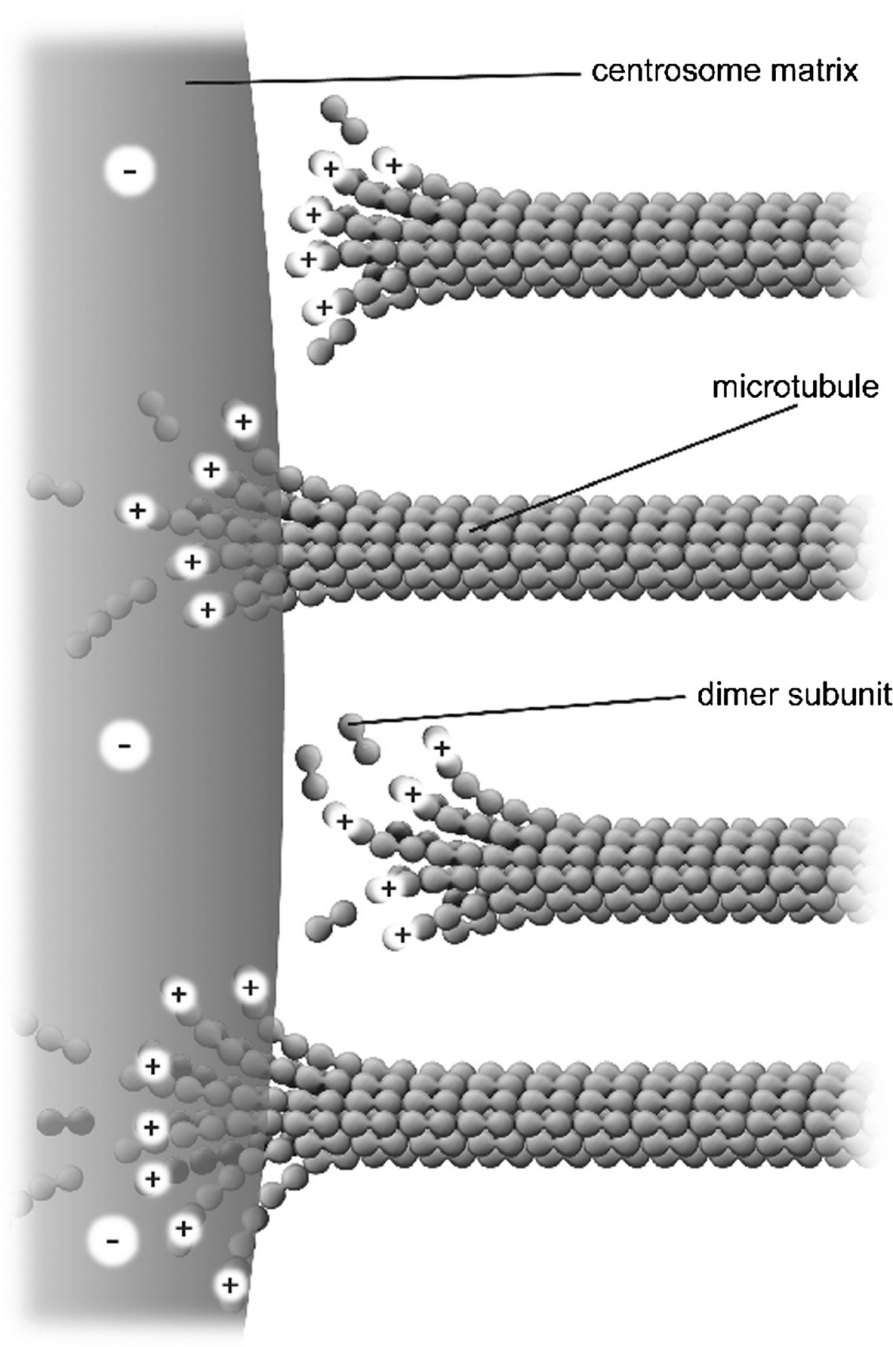
**Nanoscale electrostatic disassembly force at a centrosome.** A poleward force results from an electrostatic attraction between positively-charged microtubule free ends and an oppositely charged centrosome matrix. Only disassembling microtubules are depicted, assembling microtubules could also be momentarily attracted to a centrosome.

Based on discussions above, the net charge on the free ends of microtubules at a centrosome matrix is assumed to be positive. A *γ*-tubulin molecule, embedded in the fibrous centrosome matrix, takes the form of a ring from which a microtubule appears to emerge [32]. This would allow the electric field of the negatively-charged centrosome matrix to attract and draw positively charged ends of microtubules into the centrosome matrix. The changing electric field (and resulting force) gradient vicinal to, and across, the centrosome matrix boundary may destabilize microtubules as they approach and pass into the centrosome, as depicted in Figure 2.

Thus *γ*-tubulin rings may be regarded as forming a firmly anchored negative charge distribution through which the positively-charged, minus ends of kinetochore microtubules are drawn, generating poleward force that is associated with the observed poleward *microtubule flux*. Microtubules do not necessarily need to pass through the rings; rather, the rings provide a structurally stable, negatively-charged volume distribution attracting microtubules to, and into, the centrosome matrix.

As noted above, observations on a number of cell types have shown that disassembly of microtubules at spindle poles often accompanies chromosome poleward movement. Accordingly, within the context of the present work, force generation at spindle poles for prometaphase post-attachment, metaphase, and anaphase-A poleward chromosome motions can be attributed to an electrostatic attraction between the positively-charged free minus ends of kinetochore microtubules and a negatively-charged centrosome matrix.

The magnitude of the force produced by a non-penetrating microtubule at a centrosome matrix is calculated as follows. Since the outer diameter of a centrosome matrix is considerably larger than the diameter of a microtubule, we may model it as a large, approximately planar slab with negative surface charge density of magnitude *σ,* as depicted in Figure 2. From the well-known Debye-Hückel result for a planar, charged surface with area charge density *σ* immersed in an electrolyte [33], we have for the electrostatic potential

1 $$ \varphi (x)=\left(D\sigma /\varepsilon \right){\mathrm{e}}^{-x/D} $$

where *D* is the *Debye length*, *ε* is the cytosolic permittivity (*ε* = *k*$\varepsilon$_0_, with *k* the dielectric constant, $\varepsilon$_0_ the permittivity of free space), and *x* the distance from the surface.

The electric field *E*(*x*), obtained from the negative gradient of the electrostatic potential multiplied by the charge *q* gives the magnitude of the attractive force *F* (*x*) between the charge *q* on a dimer subunit at the free end of a protofilament and the centrosome. This results in

2 $$ \mathrm{F}\left(\mathrm{x}\right)=\mathrm{q}\ \mathrm{E}\left(\mathrm{x}\right)=\hbox{-} \mathrm{q}\left(\partial \upphi /\partial \mathrm{x}\right)=\left(\sigma \mathrm{q}/\upvarepsilon \right){\mathrm{e}}^{\hbox{-} x/D} $$

It is well established in electrochemistry that the permittivity of the first few water layers outside a charged surface is an order of magnitude smaller than that of the bulk phase [34]. The effective permittivity of water as a function of distance from a single charged surface has been determined by atomic force microscopy to increase monotonically from 4–6 $\varepsilon$_0_ at the interface to 78 $\varepsilon$_0_ at a distance of 25 nm from the interface [35]. The values of the dielectric constants *k*(*x*) at distances of 1, 2, 3, and 4 nm from a charged surface were measured to be 9, 21, 40, and 60, respectively. Layered water adhering to the net charge of proteins will significantly reduce counterion screening for small distances from the surface.

The interpolated values of *k*(*x*) for separations between charged surfaces of up to 3 nm are 5, 9, 9, and 5 for *x* = 0, 1, 2, and 3 respectively, where the charged surfaces are at *x* = 0 and *x* = 3 nm (the experimental value of *k*(*x*) at both *x* = 0 and *x* = 3 is 5; symmetry and the experimental numbers dictate the values of 9 in between.) The distance range 1 to 3 nm between charged surfaces is important for the present calculation because 1 nm may be taken as the thickness of layered water adsorbed to each charged surface [30,36], and for charged molecular surface separations up to 3 nm, counterion (*Debye*) screening would be virtually eliminated. Thus electrostatic force is increased over the distances allowed by reduced Debye screening, and is further increased (by an order of magnitude) due to an order of magnitude reduction in the dielectric constant between the charged surfaces. For brevity, separations of 0 to 3 nm (and, due to the reduced dielectric constant between charged molecular surfaces, 1 to 2 nm beyond) between charged surfaces will hereafter be designated as *critical distances/gaps*.

For critical distances, the expression for the force between a charged centrosome matrix surface at *x* = 0 and a charge *q* on the free minus end of a protofilament at a distance *x* from the surface may therefore be written

3 $$ F(x)=\sigma q/\varepsilon (x), $$

where *ε*(*x*) = *k*(*x*) $\varepsilon$_0_ is obtained from the interpolated experimental results for *k*(*x*) referred to above, $\varepsilon$_0_ = 8.85 pF/m (picoFarads per meter) and *q* is the charge on the protofilament free end. This equation may be obtained from (2) in the limit as *D* → ∞, a condition that effectively eliminates counterion screening.

Thirteen protofilaments are arranged circularly in a microtubule, with an axial shift of 0.92 nm for each protofilament as one moves around the circumference of a B lattice microtubule [15]. For comparison with experimental values, and to get a sense of the strength of the electrostatic forces, a calculation of the total disassembly force per microtubule due to protofilaments at distances of 2 and 3 nm from a centrosome is presented. The actual distribution for distances of the free ends of 13--disassembling (curling) and temporarily assembling (straight)--protofilaments would be considerably complicated, and it is probable that several protofilaments from a close microtubule interact with a centrosome matrix within critical distances at any given time. Experimental values of surface charge density *σ* for biological surfaces range from 1 to 50 mC*/*m^2^ (milliCoulombs per square meter) [37,38]. Thus, we may calculate the forces on protofilament free ends at the above distances from a centrosome matrix using the interpolated *k*(*x*) values of 9 at the 2 nm distance, and 5 at the 3 nm distance, along with a conservative value for *σ* of 20 mC*/*m^2^. Carrying out this calculation with (3), the electrostatic force on the two protofilaments sums to 148*n* pN/MT (picoNewtons per microtubule), where *q* = *n e*, with *e* equal to the magnitude of the charge on an electron and *n* the number of electron charges at the protofilament free end. Comparing this value with the experimental range of 1–74 pN/MT for the maximum tension force per microtubule [39], we have that *n* = 0.007 - 0.5 electron charges. This range compares favorably to experimental values [15,17,40], and the agreement represents a successful *ab initio* theoretical derivation of the force magnitude. Note that this calculation can be done in a number of ways dependent on specific assumptions; nonetheless, all of the justifiable calculations lead to ranges for protofilament free end charges that are well within the experimental range.

Thus we may envision a process whereby force generation from an instantaneous subset of protofilaments (at critical centrosome distances within a number of microtubules) continues with other subsets of constantly changing larger and smaller (critical) gaps, causing kinetochore microtubule bundles to move toward a centrosome matrix while doing work. Polymerization in gaps larger than the 8 nm length of tubulin dimers, along with depolymerization elsewhere, continues as overall “contact/tracking” is maintained by critical gap forces during the complex motions of mitosis. Note that polymerization in gaps slightly greater than 8 nm would be expected to place tubulin dimers close to or within critical distances for force generation. As discussed elsewhere [41,42], chromosome movements during mitosis may depend on a changing microtubule disassembly to assembly probability ratio. With an increase in this ratio (i.e., higher net disassembly rate), there will be less opportunity for polymerization since advancing microtubules can more frequently shorten centrosome matrix distances to less than 8 nm.

Electrostatic force at a centrosome due to penetrating microtubules will now be considered. Since centrosome diameters are large compared to the diameters of protofilaments, we may model the centrosome-microtubule interaction for penetrating microtubules by assuming an approximately planar slab of uniform negative charge density, with thickness *a* parallel to the *x* axis (the microtubule axis) for the outer edge of the centrosome matrix, interacting with positively-charged free ends of microtubule protofilaments, as depicted in Figure 2.

A standard result from an application of Gauss’s law [43] gives the following result for the magnitude of the electric field inside a large, uniformly charged slab

4 $$ \mathrm{E}\left(\mathrm{x}\right)=\uprho \mathrm{x}/{\varepsilon}_1 $$

where *ρ* is the volume charge density, *ε*_1_(=*k*_1_*ε*_0_) is the dielectric permittivity of the slab, and *x* = 0 at the plane of symmetry in the center of the large rectangular slab. (Note that previously in (3), *x* = 0 at the right boundary of the centrosome matrix; Figure 2.)

Employing the uniform charge relation *σ* = *ρ a*, this result may be expressed in terms of the surface charge density *σ* as

5 $$ \mathrm{E}\left(\mathrm{x}\right) = \sigma x/{\varepsilon}_1\mathrm{a} $$

The magnitude of the force on a protofilament of positive charge magnitude *q* at its free end, distance *x* from the plane of symmetry is given by

6 $$ \mathrm{F}\left(\mathrm{x}\right)=q\mathrm{E}\left(\mathrm{x}\right) = q\sigma x/{\varepsilon}_1\mathrm{a} $$

At the right boundary of the (negatively-charged) centrosome, *x* = *a/*2, *E* = −*σ/* 2 *ε*_1_, and the magnitude of the force exerted in the negative *x* (poleward) direction on a protofilament free end with positive charge of magnitude *q* located just inside the right face is *σ q/* 2 *ε*_1_.

The value of the dielectric constant *k*_1_ for a centrosome matrix has not been established. Due to an open structure that allows cytoplasmic water intrusion, the large dielectric constant of water would strongly influence the overall dielectric constant of the centrosome matrix, leading to a value that is relatively insensitive to the *dry* value. Consistent with their open structures, a cytosol-saturated centrosome matrix would be expected to have a dielectric constant that is quite large, roughly midway between the dry value and cytoplasmic water [44]. Therefore, the value for cytoplasmic water will dominate, and the calculation is relatively insensitive to the precise dry value. For simplicity, since most condensed-matter dielectric constants are between 1 and 5, an approximate conservative midpoint value *k*_1_ = 45 ((80 + 10)*/*2) will be assumed. Using *k*_1_ = 45 and the value *σ* = 20 mC*/*m^2^ in carrying out a conservative calculation with (6) for a microtubule with 6 of the 13 protofilament ends at an average distance just inside the right boundary (*x* = *a/*2) of the centrosome matrix, we find that the force on a penetrating microtubule sums to 24 *n* pN/MT. Equating this result to the experimental range 1 – 74 pN/MT [39], *n* = 0.04 - 3 electron charges, again well within the experimental range. As described above, since the calculated range of *n* falls well within the experimental range, moderate differences in *k*_1_, the geometry, and other contributing factors would not significantly affect the outcome of this calculation.

Given the electrical nature of tubulin microtubule subunits, the electric field (and therefore force) gradient within vicinal cytosol at a centrosome matrix would increase the lability of microtubule minus ends. Additionally, the field gradient across the centrosome matrix boundary can act to destabilize microtubules, thus increasing the depolymerization probability of microtubules approaching and penetrating a centrosome as force is generated, which is in agreement with experimental observations.

## Conclusions

It seems clear that cellular electrostatics involves more than traditional thinking regarding counterion screening of electric fields and the putative unimportance of the second most powerful force in nature. Rather, the evidence suggests otherwise, namely that enhanced electrostatic interactions are more robust and act over greater distances than previously thought. One consequence of this is the ability of microtubules to extend the reach of electrostatic force over cellular distances; another lies in the reduced counterion screening and dielectric constant of the cytosol between charged molecular surfaces.

Given a net negative charge on centrosomes and positive charges at microtubule minus ends, an attractive electrostatic poleward-directed force between the microtubule minus ends and centrosomes is difficult not to envisage. Calculations of electrostatic force magnitudes for penetrating and non-penetrating microtubules show that nanoscale electrostatic interactions are independently able to account for poleward force generation at cell poles. The calculated force per microtubule falls within the experimental range and represents a successful *ab initio* derivation of the force magnitude.

The present model assumes that force generation is due to both penetrating and non-penetrating microtubules. Force generation by nanoscale electrostatic non-contact interactions, primarily over critical distances, would seem essential for efficient microtubule reattachment and tracking to centrosomes throughout the complex motions during mitosis, a feature that is not explained by any current models of chromosome motility.
